# Unwelcome neighbours: Tracking the transmission of *Streptococcus equi* in the United Kingdom horse population

**DOI:** 10.1111/evj.14558

**Published:** 2025-07-20

**Authors:** Abigail A. McGlennon, Kristien L. Verheyen, J. Richard Newton, Andries van Tonder, Hayley Wilson, Julian Parkhill, Nicolas de Brauwere, Sara Frosth, Andrew S. Waller

**Affiliations:** ^1^ Royal Veterinary College Hertfordshire UK; ^2^ Department of Veterinary Medicine University of Cambridge UK; ^3^ Public Health Genomics Foundation Cambridge UK; ^4^ Redwings Horse Sanctuary Norwich UK; ^5^ Department of Animal Biosciences Swedish University of Agricultural Sciences Uppsala Sweden; ^6^ Intervacc Hägersten Sweden

**Keywords:** bacteriology, genomics, infectious disease, *Steptococcus equi*

## Abstract

**Background:**

Strangles (*Streptococcus equi* infection) remains endemic in the UK, with ~300 laboratory diagnoses annually. Sub‐clinically infected long‐term carriers are considered a key driver of endemicity. Analysing genomes of circulating strains could provide valuable transmission insights of this pathogen.

**Objectives:**

To determine the population structure and diversity of UK *S. equi* isolates and to model transmission using epidemiological and whole genome sequencing data.

**Study Design:**

Retrospective cross‐sectional epidemiological and genomic surveillance.

**Methods:**

A dated phylogenetic tree derived from 511 *S. equi* isolates collected from UK horses between 2015 and 2022 was reconstructed. Bayesian Analysis of Population Structure (BAPS) identified clusters of related genomes, while iGRAPH identified clusters of sequences appropriate for transmission analysis, performed using Transphylo.

**Results:**

BAPS identified nine groups, with 82% of strains clustering into two (McG‐BAPS3, McG‐BAPS5). A statistically significant association (*p* < 0.001) was found between the year of recovery and trends in the frequency of McG‐BAPS groups, with McG‐BAPS3 increasing and McG‐BAPS5 decreasing in prevalence over the study period. Eight transmission clusters encompassing 64% of total sequences (*n* = 286/447) underwent analysis. Sixteen direct transmission pairs were identified; 10 were between horses from different UK regions. A transmission chain extending over a 6‐month period was inferred from isolates from nine horses.

**Main Limitations:**

Bacterial strains from sub‐clinically infected carrier horses may be underrepresented due to data collection via positive laboratory diagnoses. Furthermore, a low sampling proportion relative to overall UK cases provided only a snapshot of broader, unsampled transmission events.

**Conclusions:**

The rapid change in *S. equi* population structure indicates acutely infected/recently convalesced short‐term carrier horses play a more influential role in transmission than long‐term carriers. Our work provides novel insights to our understanding of *S. equi* transmission dynamics. Transmission of genetically related strains across diverse regions suggests a real‐time sequence‐based surveillance system could inform interventions to minimise transmission.

## 1 INTRODUCTION

*Streptococcus equi* is a host‐restricted pathogen affecting equids and is the causative agent of the highly infectious bacterial disease commonly referred to as ‘strangles’.^1^ Strangles is endemic among the UK horse population and can be transmitted via direct nose‐to‐nose contact or indirectly via fomites and shared water sources.^2^ *Streptococcus equi* can be detected within the lymph nodes of infected horses within a few hours of exposure.^3^ Here, the organism stimulates an uncontrolled inflammatory response forming abscesses than can be so large as to restrict the airway, causing the death of ~1% of infected horses.^4^ Rupture of the abscesses releases infectious material into the local environment, which may be transmitted to other horses. After acute infection, incomplete drainage of purulent material from the guttural pouch arising from retropharyngeal lymph node abscess rupture, results in the establishment of persisting infection in some recovered horses that are outwardly healthy. These subclinical carriers can remain intermittently infectious for months, if not years, shedding bacteria into their local environment until they are identified and treated.^5^, ^6^ Therefore, subclinical carriers are thought to be a major factor in endemicity of strangles and *S. equi* transmission between equine premises.^7^

Controlling the spread of strangles is challenging due to incomplete uptake of biosecurity recommendations and low levels of post‐outbreak testing to identify persistent subclinical infections.^8^ Despite evidence‐based recommendations,^9^ strangles is currently not included in the World Organisation for Animal Health's (WOAH) list of diseases, infections and infestations of the horse. Additionally, in the UK there is no regulatory requirement to report outbreaks and initiate contact tracing, leading to suboptimal circumstances to investigate *S. equi* transmission nationally or internationally. The Surveillance of Equine Strangles (SES) initiative collates and reports diagnoses of strangles made across the UK^10^, ^11^ by a broad network of diagnostic laboratories that are responsible for the majority of laboratory confirmations of strangles nationally. During strangles outbreaks, clinical samples are taken for diagnostic confirmation of *S. equi*, and SES has created a biobank of *S. equi* isolates from surplus diagnostic samples supplied by the laboratory network and subjected to subsequent whole genome sequencing (WGS).^12^

Whole genome sequencing of *S. equi* strains and the use of Bayesian methods have provided a statistical framework to explore the phylogeny and population structure of *S. equi*.^9^, ^13^, ^14^, ^15^, ^16^ Within the UK, WGS of *S. equi* isolates showed that closely related strains were not limited to certain geographical locations and individual outbreaks but could be recovered from multiple regions.^12^ Bayesian methods to study and infer the transmission of infectious disease have enabled transmission dynamics to be modelled through combining genomic and epidemiological information.^17^, ^18^, ^19^, ^20^ This includes the modelling and reconstruction of pathogen phylogenetic trees which can subsequently be used to investigate transmission events between infected individuals.^21^, ^22^ The open‐source R package ‘Transphylo’ enables the analysis of partially sampled outbreaks and quantifies the likelihood of transmission between genetically related isolates through a Bayesian framework, while accounting for within‐host variation.^18^ These software features provided an unprecedented opportunity to investigate the combination of genomic and epidemiological data to examine the transmission of *S. equi* in the UK horse population.

This study aimed to determine the genetic variation and to define the transmission dynamics of circulating *S. equi* strains recovered from horses across the UK between 30 December 2015 and 14 September 2022. Using computational methods, we combined *S. equi* genomic data with clinical history from the corresponding SES laboratory reports to determine whether strains were transmitted locally or nationally and whether direct transmission links between horses could be identified. Our findings provide novel insights into the likely processes and factors underpinning the transmission of *S. equi* in the UK that will direct targeted interventions and improve outbreak management protocols, thereby helping to reduce the occurrence of strangles and the impact of outbreaks on equine premises and businesses.

## 2 METHODS

### 2.1 Study samples

A total of 511 *S. equi* bacterial isolates from UK diagnoses of *S. equi* infections were provided by participating SES laboratories between 30 December 2015 and 14 September 2022, with the exception of March 2020–June 2021. In this period, sample submissions were paused due to laboratory closures and disruption caused by the SARS‐CoV‐2 pandemic. This collection from SES includes 339 isolates from 2015 to 2019, whose genomes had been sequenced previously.^12^ The origins and details of the 511 isolates are provided in Table S1. Briefly, surplus diagnostic samples were submitted by SES laboratories to the Animal Health Trust before March 2020 (prior to its closure), and the research laboratory at the Royal Veterinary College after June 2021 following a positive diagnosis of *S. equi* infection. To be included in the study, a pure *S. equi* colony was required after culture, confirmed by sugar‐fermentation tests,^23^ upon receipt at the research laboratory, ensuring suitability for WGS. In some instances, bacterial culture was not possible due to the lag phase from sampling to receipt and therefore reduced bacterial viability or heavy contamination. Therefore, while these samples may have been submitted to the SES network, they could not be included in this study. However, our dataset includes the majority of culturable *S. equi* isolates during the study period that were processed by SES diagnostic laboratories, which cover most strangles diagnoses across the UK.

### 2.2 DNA extraction and whole genome sequencing

For 172 samples, single *S. equi* colonies were inoculated into 2 mL Todd Hewitt broth containing 30 μg/mL of hyaluronidase (Sigma Aldrich, UK) and incubated overnight at 37°C in a humidified atmosphere containing 5% CO_2_. Cultures were pelleted by centrifugation and DNA extraction completed using the Sigma GenElute™ Bacterial Genomic DNA Kit (Sigma Aldrich). Specifically for *S. equi*, 25 μL of mutanolysin reconstituted with ddH_2_O to 50 units per mL (Sigma Aldrich) was included in the Gram‐positive lysozyme solution.

DNA libraries were prepared following Illumina's Nextera XT DNA Library Preparation protocol and sequenced using an Illumina NextSeq 500 benchtop sequencer with 2 × 150 bp paired‐end reads.

### 2.3 Phylogenetic analysis

The quality of all 511 sequenced genomes was examined using the BacQC and Bactmap pipelines (doi:10.5281/zenodo.15046661, https://nf-co.re/bactmap) (Results available in Table S2). Fastq files were assembled using Snippy and the ‘snippy‐multi’ command (https://github.com/tseemann/snippy) where sequences were mapped to the reference genome *Se*4047 (Accession number: FM204883), which was recovered from a horse in the New Forest, Hampshire, UK, in 1990.^1^ A python script was used to combine the two data files produced by Snippy which then created a mapped alignment for each sample (https://github.com/francesccoll/scripts/blob/main/create_snippy_consensus.py). Aligned sequences were then concatenated into one multi‐fasta alignment.

For phylogenetic reconstruction, the mobile genetic elements ICESe1, ICESe2, prophages φSeq1 to φSeq4, and regions SEQ_2017 (SeM) and SEQ_0933 (SzPSe) were removed using a Sanger‐Pathogens script (https://github.com/sanger-pathogens/remove_blocks_from_aln). This was due to their confounding effects on phylogenetic interpretation and known high density of SNP homoplasies across the SeM and SzPSe regions.^15^ Using Gubbins,^24^ and *Se*4047 as a reference, loci within the core alignment that contained increased densities of base substitutions likely to have been introduced by recombination were identified^21^ and these were masked using the python function ‘maskrc‐svg.py’ (https://github.com/kwongj/maskrc-svg). The number of sites containing a single nucleotide polymorphism over all sequences within the alignment was identified using the bioinformatics tool SNP‐sites.^25^ Following this, a consensus tree was reconstructed using IQ‐TREE^26^, ^27^ with ultrafast bootstrapping24 (−bb 1000), details of the constant sites (−fconst) and a general time reversible (GTR) model. Bayesian analysis of population structure (BAPS) was undertaken using the R package ‘fastBAPS’ (Fast Bayesian Analysis of Population Structure)^14^ on the consensus tree and alignment reconstructed in IQ‐TREE.

For Bayesian Evolutionary Analysis by Sampling Trees (BEAST) reconstruction, temporal signal (the correlation between measurable genomic evolution and time over a given period, where sequences can be taken at different points in time, and there are a statistically significant number of genetic differences^28^) was determined by examining the correlation of the root‐to‐tip distances against sampling date using TempEst.^29^ A bootstrap analysis was run 1000 times to further determine the significance of a correlation between root‐to‐tip distance and sample collection date. A maximum clade credibility tree was then reconstructed in BEAST2—version 2.6.3,^30^ using the following methodology. First, a control file was designed in BEAUti2 (Bayesian Evolutionary Analysis Utility) to configure sampling dates to the multi‐fasta alignment, specify the site and clock models to use, and alter the priors used for the analysis. Configuration followed recommendations by Harris et al.^15^ where a relaxed exponential clock model and coalescent Bayesian skyline population model were used. Three independent BEAST runs of 100,000,000 Markov chain Monte Carlo (MCMC) iterations with sampling every 10,000 iterations were completed. After 100,000,000 MCMC iterations, convergence was assessed using the effective sample size (ESS) parameters for each triplicate run in Tracer version 1.7.2^31^ where ESS values ≥200 were deemed to have converged successfully. Each triplicate run was combined in LogCombiner and a maximum clade credibility (MCC) tree created in TreeAnnotator.^32^ A date‐randomisation test (DRT) was run using the package ‘TipDatingBeast’^33^; 10 replicate BEAUti input xml files were created containing randomised permutations of the sampling times and run as individual BEAST analyses. The output parameter estimates were compared between the original data set and the randomised date sets. The DRT was deemed successful if, when compared, the 95% highest posterior density (HPD) of the substitution rate estimated for the original data set did not overlap with the randomised datasets.

### 2.4 Transmission analysis

Excluding 64 samples that were sequential to the first chronologically recovered isolate from the same horse (identified via matching horse name and veterinary practice name, linked as follow up veterinary investigation/outbreak clearance testing samples after initial positive diagnosis), a total of 447 *S. equi* whole genome sequences contributed to the reconstruction of a MCC phylogenetic tree following the methods outlined above. Clusters of sequences that were appropriate candidates for transmission analysis were identified using the iGRAPH R package,^34^ which plotted a network of the 447 sequences determined by a threshold of five pairwise single nucleotide polymorphisms (SNPs) between any two core genome samples, calculated using the R package pairsnp v1.0 (https://github.com/gtonkinhill/pairsnp). Pairwise SNP distance thresholds of 1, 2, 3, 10, 15, and 20 were also calculated and plotted (Figure S1); however, five pairwise SNPs was deemed most appropriate for cluster selection, due to the clear distinguished groupings of sequences compared with other thresholds (1–3, 10, 15 and 20) that were tested. To enable sufficient resolution for transmission analysis, clusters with fewer than 10 sequences each were not included. Bayesian inference of transmission for eligible clusters was undertaken using Transphylo.^35^

#### 2.4.1 Testing Transphylo prior parameters

To account for the diverse clinical scenarios of strangles, it was essential to evaluate the impact of varying prior parameters in the transmission model before conducting the final analyses. Therefore, testing of the prior assumptions ‘generation time’ (i.e., time between infection to onward transmission) and ‘sampling time’ (i.e., the time between infection and sampling) was undertaken to assess their effect on Markov chain Monte Carlo (MCMC) convergence and the transmission inference parameters used by Transphylo. Specifically, we sought to assess how these adjustments, representing different clinical presentations of strangles, affected model performance and its alignment with the genomic data in our sequences. To do so, the mean and standard deviation for the Gamma distribution representing both were tested using a cluster of 16 sequences across three different scenarios, acute transmission (13 ± 5 days), between outbreak transmission (42 ± 10 days) and transmission from long‐term carriers (182 ± 30 days) (Table S3). This small cluster was identified within the iGRAPH clustering investigations and is ‘Cluster five’ in the main analyses. It was selected for testing due to the small sample size and therefore a relatively quick computational processing time, allowing for efficient evaluation of a variety of clinical scenarios.

#### 2.4.2 Running the transmission inference

For each cluster, transmission inference was performed in triplicate over 10^6^ MCMC iterations, using the same prior distribution parameters for w.mean, w.std, ws.mean, and ws.std (gamma = 0.115, scale = 0.027), corresponding to a combined generation and infection time of 42 ± 10 days. The sampling proportion was set as 0.5 (Transphylo's default).

Assessment of the convergence of MCMC simulations is required before exploring the results of transmission inference as successful convergence indicates that the model has reached a stable state representing the true posterior distribution of the model parameters allowing for reliable inferences to be made. If the model has not converged successfully it means the model is not accurately capturing the posterior distribution leading to unreliable results. Therefore, once inference was complete, a burn‐in of 10% for each inference was set before assessing MCMC convergence using the R package Coda.^36^ For each triplicate run, successful convergence was indicated when the parameters, ‘offspring distribution’ (off.r), ‘sampling probability’ (pi) and ‘within‐host diversity’ (neg),^35^ had Gelman and Rubin's (GR) Convergence Diagnostics of <1.2^37^ and ESS values >100. The posterior probability of transmission events between sampled individuals was calculated using the first triplicate run, where a probability of ≥0.5 indicated a plausible transmission event. The distances between sampled individuals within the input phylogeny were also computed, and a consensus transmission tree reconstructed. Where possible, transmission events were further considered and validated using epidemiological data available from the SES database which was provided by submitting veterinary surgeons when requesting laboratory confirmation of *S. equi* infection. This included geographical location (details in statistical methods) and where provided, clinical history including reason for sampling or an indication of infection status ‘acute’, ‘post‐infection screening’ or ‘subclinical’—although the duration of infection/chronicity was not overtly reported. For the purposes of this study and further discussion Table 1 summarises definitions used to define subclinical infection and to distinguish short‐term *S. equi* carriage and long‐term *S. equi* carriage.

**TABLE 1:** Defining the terminology surrounding strangles (*Streptococcus equi* infection) carriage and subclinical infection, and to distinguish recently convalesced subclinical short‐term carriers and long‐term carriers.

Terminology	Definition
Sub‐clinically infected	A horse with a positive laboratory diagnosis of *S. equi* infection reported to the Surveillance of Equine Strangles Network that *was not exhibiting clinical signs of infection* at the time of sampling. Categorised by information provided by the submitting veterinary surgeon including clinical notes or reason for sampling (post‐infection screening, pre/post movement, post‐positive indirect enzyme‐linked immunosorbent assay [iELISA], in contact).
Recently convalesced, subclinical short‐term carrier	Horses that have had clinical strangles infection and although appear visually to have recovered from strangles, still harbour *S. equi* within their guttural pouches 4 weeks after the cessation of their clinical signs. These horses should be identified as part of post‐infection screening to prevent them from becoming long‐term carriers. If no post‐infection screening is conducted, the infection remains, and these horses can transmit *S. equi* to new horses while transitioning into long‐term carriers.
Long‐term carrier	Horses that once exhibited clinical infection with *S. equi* but failed to clear the guttural pouch empyema during convalescence. These horses would have likely not undergone post‐infection screening, therefore have remained infected for months, if not years, and are able to sporadically shed *S. equi* and infect other horses.

Abbreviation: iELISA, indirect enzyme‐linked immunosorbent assay.

### 2.5 Statistical and spatial analysis

Descriptive outputs and statistical analyses were compiled and undertaken using R version 4.2.3^38^ through RStudio version ‘2023.06.2 + 561’.^39^ After removal of 64 sequences from sequential samples from the same animal (*n* = 64), fastBAPS proportions were summarised individually and within each year and presented as percentages with 95% confidence intervals (95% CI) as well as graphically. A Pearson's Chi‐squared test of independence was performed to examine the relationship between year and the most prevalent groups. To examine the relationship between year of recovery and the odds of identifying a given group over the study period, univariable ordinary logistic regression analyses were conducted separately for the two most prevalent fastBAPS groups, McG‐BAPS3 and McG‐BAPS5. Year of recovery was the only independent variable, treated as a fixed effect with six ordered independent categories representing whole years. After graphical assessment of the linear nature of the log natural coefficient values the variable ‘year’ was treated as continuous enabling an overall OR to be calculated via ordinary logistic regression. For all statistical analyses, the level of statistical significance was set at *p* < 0.05.

Where applicable, maps were produced using QGIS and regions of the UK were classified by Nomenclature of Territorial Units for Statistics Three (NUTS3), a geocode standard for referencing the subdivisions of countries for statistical purposes, with some minor amendments to include the Isle of Man and the Channel Islands within the UK spatial framework. As horse locations were not available for *S. equi* sequences, locations were based on the address of the submitting veterinary practice and mapped using latitude and longitude point coordinates to the postcode, enabling broad geographical distribution data to be presented consistently. The location of four outbreaks that were managed by a single veterinary surgeon were mapped to the approximate location of their holding paddocks in Dartmoor, provided by the veterinary surgeon.

## 3 RESULTS

### 3.1 Population structure and diversity of UK *S. equi* strains

**FIGURE 1:**
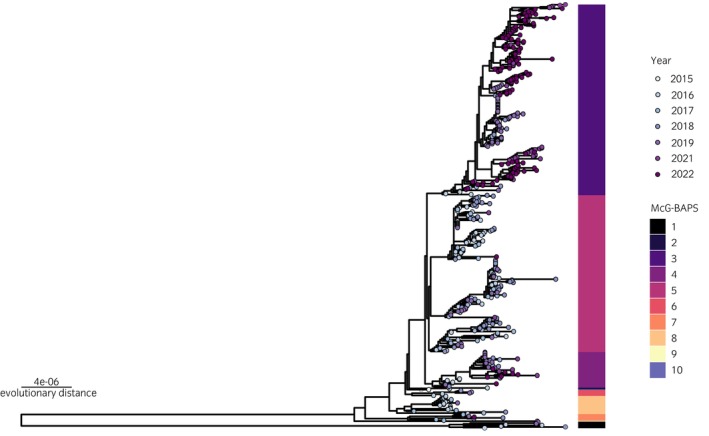
Midpoint routed maximum likelihood tree of 511 *S. equi* isolates recovered between 30 December 2015 and 14 September 2022 with McG‐BAPS groupings visualised in the right‐hand bar. The scale bar relates to evolutionary change along the horizontal branch lengths and indicates the number of SNPs difference between sequences. Coloured circles indicate the year in which the sample was recovered; the darker the shade, the more recently the sample was collected. A gap is present in 2020 due to the SARS‐CoV‐2 pandemic halting sample acquisition. Population groupings were determined by fast hierarchical Bayesian analysis of population structure (fastBAPS).^14^

A temporal signal in 511 *S. equi* sequences recovered from horses across the UK between the 30 December 2015 and 14 September 2022 was evident, with a root‐to‐tip correlation *R* ^2^ of 0.489 and a significant date‐tip permutation test (*p* < 0.001 from 1000 permutations), and a midpoint routed maximum likelihood phylogenetic tree was constructed (Figure 1). The BEAST analysis calculated the mean substitution rate per core genome site per year as 5.86 × 10^−7^ (95% HPD: 5.36–7.41 × 10^−7^) and time to most recent common ancestor (tMRCA) estimated as a median of 1963 (95% HPD: 1930–1988) (Figure S2). Ten fastBAPS groups, referred to as ‘McG‐BAPS’ hereafter, were identified (Table 2).

**TABLE 2:** Summary of descriptive data for population structure groupings with 95% confidence intervals (95% CI) and multilocus sequence type (MLST) for447 *S. equi* isolates recovered from separate UK horses between 30 December 2015 and 14 September 2022.

FastBAPS group	Total number	Percentage (%)	Percentage 95% CI (%)	MLST^a^
McG‐BAPS3	199	44.5	39.9–49.3	151
McG‐BAPS5	167	37.4	32.9–42	151
McG‐BAPS4	40	8.9	6.5–12.1	151
McG‐BAPS8	20	4.5	2.8–6.9	179
McG‐BAPS7	7	1.6	0.7–3.3	179
McG‐BAPS1	6	1.3	0.5–3	179
McG‐BAPS6	5	1.1	0.4–2.7	151
McG‐BAPS2	2	0.2	0–1.4	151
McG‐BAPS9	1	0.2	0–1.4	179
McG‐BAPS10	1	0.2	0–1.4	151

*Note*: Population groupings were determined by fast hierarchical Bayesian analysis of population structure (fastBAPS).^14^

^a^ Multilocus sequence type.

**FIGURE 2:**
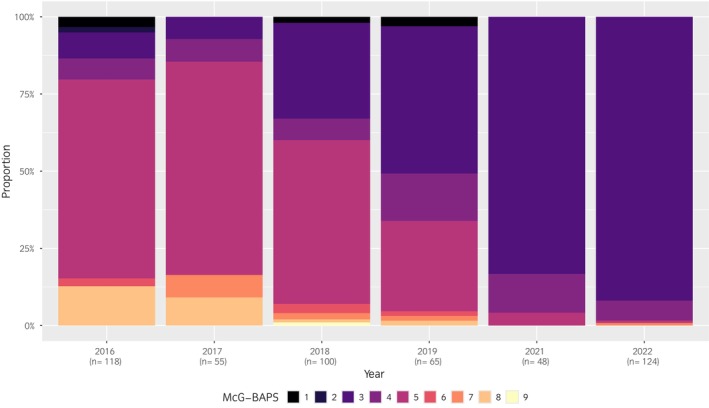
Stacked bar plot of McG‐BAPS groups for 446 *S. equi* sequences recovered from separate UK horses between 4 January 2016 and 14 September 2022. Population groupings were determined by fast hierarchical Bayesian analysis of population structure (fastBAPS).^14^ A gap is present in 2020 due to the SARS‐CoV‐2 pandemic halting sample acquisition.

FastBAPS groups categorised by year are shown alongside the phylogeny in Figure 1 and, after exclusion of the single sequence from 2015 (McG‐BAPS10) and sequential samples from the same horse (*n* = 64), graphically in Figure 2. There was a significant association between year of recovery and the distribution of relative proportions of McG‐BAPS groups, over the period (*X* ^2^(5) = 200.55, *p* < 0.001).

Results from logistic regression modelling are presented in Table S4. Between 2016 and 2022, the recovery of MCG‐BAPS3 strains increased yearly, with a statistically significant increase from 2018 onwards (*p* < 0.001). During the study, MCG‐BAPS3 coefficient values showed an increasing linear trend, with the overall OR value of 2.3 applied multiplicatively each year. The increase in McG‐BAPS3 strains corresponded with a reduction in the recovery of McG‐BAPS5 strains, with a statistically significant decrease from 2019 onwards (*p* < 0.001). During the study, MCG‐BAPS5 coefficient values showed a decreasing linear trend, with the overall OR value of 0.5 applied multiplicatively each year. The increasing recovery of McG‐BAPS3 and decrease of McG‐BAPS5 *S. equi* strains thereby illustrated the active replacement of *S. equi* genotypes within a relatively short period of 7 years from 2016 to 2022. Transmission analysis of strains within each McG‐BAPS group, broken down into smaller clusters based on their genetic relatedness, was then undertaken.

### 3.2 Transmission analysis cluster selection

**FIGURE 3:**
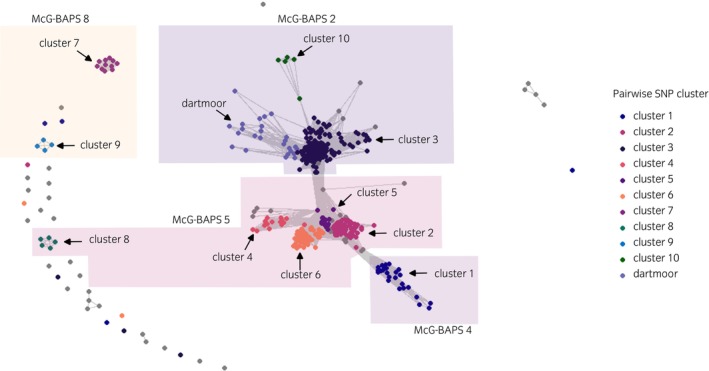
Network plot with clusters of the pairwise single nucleotide polymorphism (SNP) distances between the 447 *S. equi* sequences recovered from individual horses between 30 December 2015 and 14 September 2022 across the United Kingdom with the pairwise SNP distance threshold set as ‘five’ between sequences. Clusters have been highlighted relating to their corresponding McG‐BAPS groups.

The threshold distance of five pairwise SNPs provided clear clusters of *S. equi* sequences (Figure 3), and visual inspection of these overlaid on the phylogenetic tree enabled the identification of 10 clusters and one targeted subcluster (Dartmoor) to be selected. Eight clusters were comprised of sequences ranging in number from 11 to 177 (Table 3), comprising 64% of the total sequences (*n* = 286/447). Within cluster three, a subcluster (Dartmoor) containing 25 *S. equi* sequences was selected for separate transmission inference. The detailed outbreak management and clinical history of this subcluster made it a suitable candidate to validate transmission inference outputs.

**TABLE 3:** Summary information for the seven clusters and one subcluster of *S. equi* sequences eligible for transmission analysis, including the number of sequences within the cluster, the date range for when samples were recovered and from how many regions of the UK that samples were recovered as classified by Nomenclature of Territorial Units for Statistics Three (NUTS3).

Cluster	Sequences (*n*)	Date range (dd/mm/yyyy)	Number of UK NUTS3 regions recovered from
Cluster one	29	11/10/2016–27/07/2022	19
Cluster two	71	13/01/2016–17/06/2022	33
Cluster three^a^	177	21/08/2017–14/09/2022	51
Dartmoor (cluster three subgroup)	25	01/10/2021–31/08/2022	1
Cluster four	15	03/01/2017–20/12/2018	10
Cluster five	16	12/01/2016–20/05/2019	11
Cluster six	57	04/01/2016–30/11/2021	27
Cluster seven	11	25/07/2016–26/02/2019	7

^a^ Including the 25 Dartmoor sequences.

### 3.3 Transmission analysis outcomes

#### 3.3.1 Transphylo parameter testing

Results from the preliminary testing of generation and sampling time scenarios are presented in Table S5. Briefly, the acute transmission and between outbreak transmission scenario tests converged successfully. However, the transmission from long‐term carriers scenario did not. For subsequent analysis, the generation and sampling time of 42 ± 10 days (accounting for an average of 2 weeks of active infection followed by 4 weeks of convalescence after the cessation of clinical signs) was deemed most appropriate given the majority of isolates were collected from differing outbreaks rather than mass screening from closed outbreaks on one premises.

#### 3.3.2 Transmission outputs

Three of the eight selected clusters (one, two, and four) showed non‐convergence, while five clusters (three, Dartmoor subcluster, five, six, and seven) showed successful convergence and were deemed acceptable for further analysis (Table S6). The total number of hosts within each cluster's transmission inference (sampled and unsampled), the calculated sampling proportion, and the number of plausible direct transmission pairs are summarised in Table 4.

**TABLE 4:** The total number of hosts within each cluster's transmission inference (sampled and unsampled), the estimated sampling proportion and the number of plausible transmission pairs identified using the R package Transphylo.^35^

	Cluster three	Dartmoor (cluster three subgroup)	Cluster five	Cluster six	Cluster seven
Total hosts	960	165	142	354	61
Sampled cases	177	25	16	57	11
Unsampled cases	783	140	126	297	50
Sampling proportion	18.4%	15.1%	11.2%	16.1%	18.0%
Plausible transmission pairs	13	1	0	3	0
Plausible pair percentage	1.3%	0.6%	0.0%	0.8%	0.0%

*Note*: Transmission inference was based on the prior assumptions ‘generation time’ (i.e., the time between infection to onward transmission) and ‘infection time’ (i.e., the time between infection and sampling) being 42 days ±10.

Sixteen plausible direct transmission pairs were identified within two clusters (Table 5). Nearly two‐thirds of transmission pairs were identified between horses from different regions (*n* = 10, 62.5%, 95% CI 35%, 85%), the remaining pairs were between horses within the same region (*n* = 6, 38%, 95% CI 15%, 65%).

**TABLE 5:** Plausible transmission pairs identified, their posterior probability, days between sampling dates and whether transmission occurred within or between UK NUTS3 regions.

Pair	Transmission cluster	Direction	ID	Date sampled	Reason for sampling	Posterior probability	Days between sampling dates	Within or between regions?
Pair 1	3	Transmitter	S389	22/10/2018	Strangles suspected	0.572	4	Within
		Recipient	S396	26/10/2018	Post‐seropositive iELISA			
Pair 2	3	Transmitter	S389	22/10/2018	Strangles suspected	1	24	Between
		Recipient	S382	15/11/2018	Post‐infection screening			
Pair 3	3	Transmitter	S402	06/11/2018	Undefined	0.82	30	Between
		Recipient	S172	06/12/2018	Clinically ill			
Pair 4	3	Transmitter	S393	26/11/2018	Clinically ill	0.512	2	Between
		Recipient	S163	28/11/2018	Clinically ill			
Pair 5	3	Transmitter	S379	02/11/2018	Clinically ill	0.596	49	Between
		Recipient	S212	21/12/2018	Strangles suspected			
Pair 6	3	Transmitter	S172	06/12/2018	Clinically ill	0.901	28	Between
		Recipient	S215	03/01/2019	Clinically ill			
Pair 7	3	Transmitter	S172	06/12/2018	Clinically ill	0.884	36	Between
		Recipient	S183	11/01/2019	Strangles suspected			
Pair 8	3	Transmitter	S215	03/01/2019	Clinically ill	0.748	9	Between
		Recipient	S126	12/01/2019	Undefined			
Pair 9	3	Transmitter	S056	29/06/2021	Strangles suspected	0.926	43	Within
		Recipient	S061	11/08/2021	Undefined			
Pair 10	3	Transmitter	S063	20/08/2021	Strangles suspected	0.649	6	Between
		Recipient	S064	26/08/2021	Strangles suspected			
Pair 11^a^	3	Transmitter	S072	01/10/2021	Post‐infection screening	0.534	0^b^	Within
		Recipient	S075	01/10/2021	Post‐infection screening			
Pair 11^a^	Dartmoor	Transmitter	S075	01/10/2021	Post‐infection screening	0.577	0^b^	Within
		Recipient	S072	01/10/2021	Post‐infection screening			
Pair 12	3	Transmitter	S490	17/05/2022	Clinically ill	0.572	29	Between
		Recipient	S466	15/06/2022	Undefined			
Pair 13	3	Transmitter	S510	08/07/2022	Pre/post‐movement	0.53	27	Within
		Recipient	S476	04/08/2022	Clinically ill			
Pair 14	6	Transmitter	S238	09/05/2016	Post‐infection screening	0.988	28	Between
		Recipient	S272	06/06/2016	Undefined			
Pair 15	6	Transmitter	S449	28/12/2018	Clinically ill	0.783	12	Between
		Recipient	S117	09/01/2019	Respiratory‐infection screening			
Pair 16	6	Transmitter	S106	07/01/2019	Strangles suspected	0.635	55	Within
		Recipient	S410	03/03/2019	Undefined			

*Note*: Transmission inference was based on the prior assumption that combined ‘generation time’ (i.e., the time between infection to onward transmission) and ‘infection time’ (i.e., the time between infection and sampling) was 42 days ±10.

Abbreviation: iELISA, indirect enzyme‐linked immunoassay.

^a^ Analysed in two independent clusters but identified as a transmission pair in both.

^b^ Sampled on the same day.

Within cluster three, two horses (S389 and S172) were detected as transmitting to two separate recipients each. Where data were available (10/11 horses), six ‘transmitters’ had clinical signs at the time of sampling, including pyrexia, nasal discharge, lymph node swelling, inappetence and lethargy. Two presented with only abscessation (S389 and S063); one was sampled as part of post‐infection screening (S072), and another underwent pre/post movement screening and was reported to have nasal discharge at the time of sampling (S510).

**FIGURE 4:**
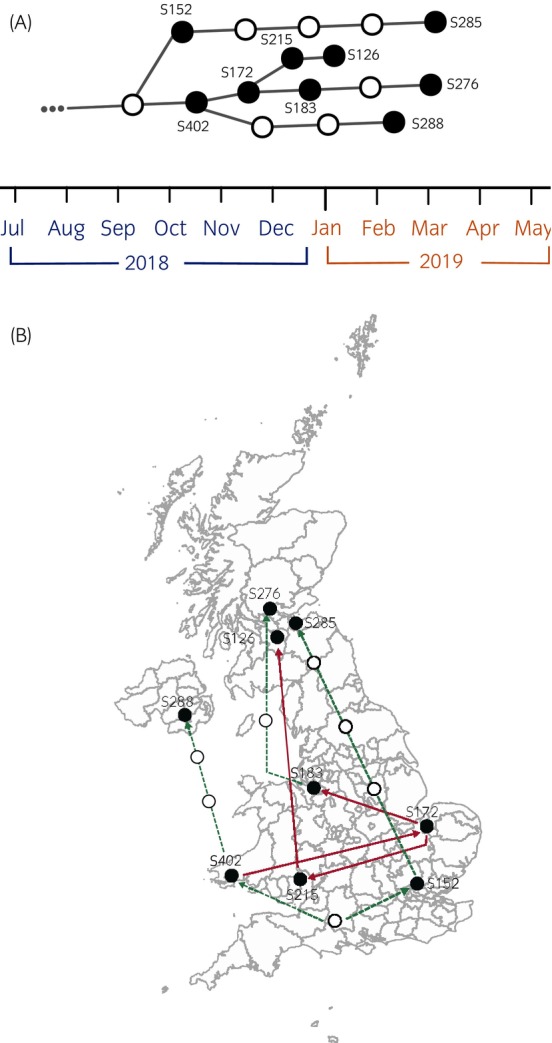
(A) Reconstructed consensus transmission tree for a group of nine sampled horses indicating a chain of transmission events across a 6‐month period in 2018 and 2019. Black circles indicate sequences derived from sampled infections, while white circles represent unsampled infections. The timeframe at the bottom of the tree represents the approximate inferred transmission timeline which might have included periods of time before sampling occurred and confirmed *S. equi* infection. (B) Mapped sequences (black circles) based on their submitting veterinary practice location. Red lines indicate direct transmission starting with S402 and following the direction of the arrow heads, green dashed lines indicate transmission links separated by unsampled cases (white circles are not plotted to accurate geographical location). Transmission inference was based on the prior assumptions that combined ‘generation time’ (i.e., time between infection to onwards transmission) and ‘infection time’ (i.e., the time between infection and sampling) was 42 days ±10.

Evidence of an onward chain of transmission among a group of nine sampled horses in cluster three was observed where plausible transmission events were identified between five horses (Figure 4A). Samples associated with this transmission chain were recovered from England, Scotland, Wales, and Northern Ireland over 6 months between October 2018 and March 2019 (Figure 4B).

The remaining three plausible transmission pairs were identified within cluster six (Table 5). Where clinical history was available (4/6 horses), S238 was previously sampled (no isolate available) 47 days before the sampling event reported here and had lymph node swelling, nasal discharge, and coughing reported at the time of first sampling. Animals S449 and S117 were reported to have had nasal discharge, and S106 had nasal discharge and pyrexia at the time of sampling.

**FIGURE 5:**
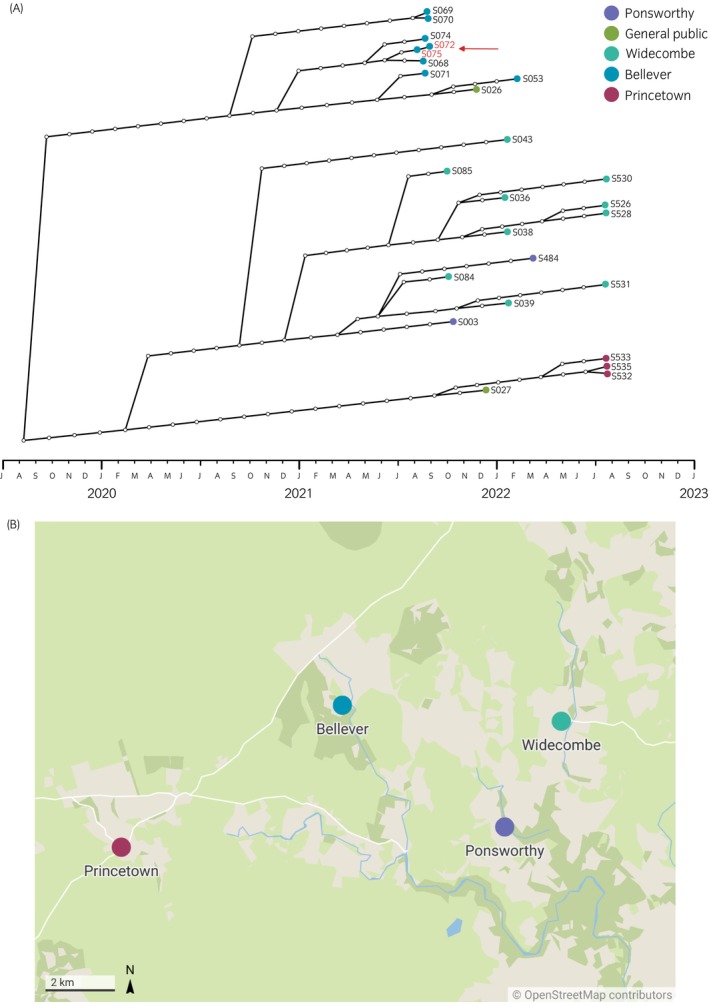
(A) Reconstructed consensus transmission tree of the Dartmoor cluster comprised of 25 *S. equi* sequences. Transmission inference was based on the prior assumptions that combined ‘generation time’ (i.e., the time between infection to onward transmission) and ‘infection time’ (i.e., the time between infection and sampling) was 42 days ±10. Coloured circles represent sampled horses and their sampling locations, and white circles are unsampled horses. ‘General public’ refers to horses and ponies not kept in semi‐feral conditions on Dartmoor. The plausible transmission pair is highlighted in red. The timeframe at the bottom of the tree represents the approximate inferred transmission timeline which might have included periods of time before sampling occurred and confirmed *S. equi* infection. (B) Map depicting the locations of the four human settlements of Bellever, Widecombe, Ponsworthy, and Princetown on Dartmoor close to separate outbreaks among four groups of semi‐feral ponies under private veterinary care (map courtesy of Google Maps).

Of the 25 sequences in the Dartmoor cluster, one plausible transmission pair was identified (Table 5 and Figure 5). Management history confirmed these ponies were part of the same herd, kept in the same quarantine paddock. Before the outbreak, they were enclosed in an area of moorland for the breeding season. In the months before the outbreak, a neighbouring farmer moved potentially infected ponies (veterinary confirmation of infection/sampling was not available) into an adjacent field, separated by a stone wall, allowing direct nose‐to‐nose contact, and clinical signs appeared in the sampled ponies 3 weeks later (Ms. Butterfield, personal communication, 2021). Acute infection was first reported in February 2021, but diagnostic sampling did not begin until October 2021. The transmission pair was also identified by analysis of cluster three, although the inferred direction of transmission differed (Dartmoor: S075 → S072, posterior probability 0.57; Cluster three: S072 → S075, posterior probability 0.53). Due to the system of management and sampling occurring on the same date, the true direction of the transmission remains unclear, but epidemiological data support a direct transmission link between the two ponies.

Four outbreaks on Dartmoor were sampled within this subcluster from different locations, based on their relative proximity to human settlements on the moor: Bellever, Widecombe, Ponsworthy, and Princetown. Isolates from Bellever and Princetown were geographically restricted to their holding paddocks, while sequences from Widecombe and Ponsworthy showed closely related sub‐strains circulating in both areas (Figure 5). Two sequences were from ponies not within the semi‐feral herds and were under general veterinary care across Dartmoor. Outbreak information available for S026 indicated this pony was kept approximately 16 miles (~26 km) from the location of Bellever and three horses from Devon were moved to the premises in the 2 weeks before the outbreak began. Outbreak information for S027 confirmed this animal was a Dartmoor pony, which was knowingly purchased while infected with strangles.

## 4 DISCUSSION

This study aimed to determine the genetic variation and to define the transmission dynamics of circulating *S. equi* strains recovered from horses across the UK between 30 December 2015 and 14 September 2022. By analysing the population structure and transmission of these isolates over this period, we sought to determine whether strains were undergoing a population replacement and whether strains were being transmitted locally or nationally, and if direct transmission links between horses could be identified. Our analysis sheds new light on the likely processes and factors that underpin the transmission of *S. equi* in the UK horse population, which can be used to direct enhanced intervention and disease prevention measures.

Throughout this study period, the primary collaborating laboratories, and therefore the provision of clinical samples, remained relatively consistent, with the exception of the closure of the Animal Health Trust (AHT). Laboratories were recruited throughout the period; however, most samples came from either the AHT prior to its closure or from three laboratories that were enrolled between 2018 and 2020. It is worth noting that the AHT laboratory caseload would have been redistributed to the other laboratories contributing to the SES scheme, so even the closure of this major laboratory for strangles diagnoses is unlikely to have significantly influenced the scheme. This consistency in sample provision underscores that the observed changes in the proportions of McG‐BAPS‐5 and McG‐BAPS‐3 strains likely reflect evolutionary trends rather than variations in sample supply over the study period.

### 4.1 Population replacements of *S. equi* occurred prior to and between 2016 and 2022

The median estimated tMRCA of UK isolates of 1963 (95% HPD: 1930–1988) and overlap in the 95% HPDs of Harris et al.^15^ (1909, 95% HPD 1819–1946) agrees with the large global population replacement suggested to have occurred in the early 20th century.^15^ The mean substitution rate per core genome site per year reported here of 5.86 × 10^−7^ (95% HPD: 5.36–7.41 × 10^−7^) is similar to that previously reported, 5.22 × 10^−7^ (95% HPD: 4.04–6.51 × 10^−7^) (15) indicating that overall *S. equi* accumulates genetic variation at a slower rate than other streptococcal species including *Streptococcus pneumoniae* (1.57 × 10^−6^)^40^ and *Streptococcus pyogenes* (1.1 × 10^−6^).^41^

Phylogenetic reconstruction of UK‐based *S. equi* isolates revealed that the collection of sequences clustered into two main clades, a smaller clade (*n* = 8) and a much larger clade comprising the remaining 503 sequences. Genomic variation between the two clades and the low number of samples within the top clade support the observation that the older strains recovered in our study were already declining in prevalence during the later part of our sampling period. This is consistent with broader reports describing a decline of one subtype (described previously as SeM‐6) of *S. equi* across Europe, coinciding with the rise of a second subtype (described previously as SeM‐9) becoming more dominant from 2010 onwards.^9^, ^12^ The population structure of international *S. equi* genome collections has been examined previously,^9^, ^42^ to which UK sequences from this study have contributed (Table S1). A total of 339 sequences presented here were included in analysis by Mitchell et al.,^9^ with sequences categorised into two of six BAPS groups, Mitchell‐2 (*n* = 331, 97.6%) and Mitchell‐5 (*n* = 8, 2.4%). Furthermore, a total of 424 sequences presented here were included in recent work by Wilson et al.^42^ with sequences categorised into 2 of 10 BAPS groups, Wilson‐7 (*n* = 416, 98.1%) and Wilson‐4 (*n* = 8, 1.9%). Notably, the eight sequences that are in groups Mitchell‐5 and Wilson‐4 are the same sequences within each independent analysis. A limitation to the fastBAPS software is that analyses are always independent; therefore, the ‘BAPS’ numbering system will not be the same between these three pieces of work. However, the consistency of the UK sequences grouping into two BAPS groups in both international collections highlights the close genetic relatedness of UK *S. equi* strains. Independent examination further showed how the UK population was categorised into 10 unique population groups with two predominant groups, McG‐BAPS3 and McG‐BAPS5, circulating in UK horses.

When categorised by yearly frequency, opposing gradient changes in the prevalences of McG‐BAPS5 and McG‐BAPS3 were observed with a significant increase of McG‐BAPS3 and decrease of McG‐BAPS5 during the 7‐year study period (2016–2022), consistent with a discernible *S. equi* population replacement. The persistence of strangles outbreaks (endemicity) has been reported to be supported by subclinical long‐term carrier horses. Carriers are typically horses that have passed the usual convalescence period of 4 weeks, which have failed to clear guttural pouch *S. equi* infection, but have not been identified through post‐infection screening and therefore remain infected for months, if not years.^5^, ^43^ However, the rapidly accumulated year‐on‐year changes in the relative frequencies of two McG‐BAPS groups observed during this study period suggest that acute infection, perhaps combined with limited adoption of biosecurity and hygiene measures by horse owners, is likely to be a more important driver of strangles transmission in the UK than previously thought. We hypothesise that the rapid population replacement across the UK (since 2016) was associated with a significant strain fitness advantage, along with rapid transmission of the new cluster and would not be consistent with long‐term carriers acting as the main source of new strangles outbreaks. This spread may have been driven by recently convalesced subclinical short‐term carrier horses (typically horses in 4 weeks after the period after the cessation of clinical signs of disease, but still harbouring *S. equi* as abscess material drains from the guttural pouches and nasopharynx) returning to normal activity/travel on the assumption of non‐infectivity, rather than following laboratory confirmation of freedom from infection. Therefore, further investigation into the adoption of outbreak management and post‐infection screening protocols undertaken by horse owners is strongly indicated, with these measures needing to be promoted by veterinary surgeons managing outbreaks. Consistent with the findings and inferences on transmission from this project, modelling of opportunities for *S. equi* transmission from long‐term carrier horses in the Netherlands estimated that these horses may be 20 times less infectious than horses with clinical strangles.^44^ Within our study, if long‐term carrier horses were driving endemicity, the change of population structure would be expected to be slower as older strains would continue to be passed from horse to horse, effectively re‐seeding infection and maintaining transmission chains, which was not observed here. It is still important to acknowledge that, while our study underscores a renewed focus on the management and treatment of recently convalesced subclinical short‐term carrier horses, unidentified carrier horses with medium‐ to long‐term infections are still potentially a factor in the transmission of *S. equi*. Consequently, the identification of these animals, or more reactively, the prevention of horses becoming long‐term carriers through post‐infection screening and clearance, remains crucial to limiting the spread of strangles within the UK's equine population, which within our study was concerningly widespread.

### 4.2 Concerning widespread transmission of *S. equi* across the UK

Combining genomic and epidemiological information made it possible to investigate *S. equi* transmission between horses, albeit with limitations in the extent of the accompanying epidemiological data and low sampling proportion estimated across the clusters ranging between 11% and 18%. The large number of unsampled horses calculated by Transphylo likely relates to the realistically small number of horses that receive a laboratory confirmation of *S. equi* infection,^11^ particularly once strangles has been confirmed on a premises and additional horses with clinical signs are no longer sampled because infection is assumed, therefore, limiting bacterial isolates available for future research. Encouragingly, where epidemiological information was available via information supplied by submitting veterinary surgeons, it usually supported Transphylo's results. This transmission analysis provides novel contributions to our understanding of the infection dynamics of an endemic equine disease, revealing how geographically dispersed and temporally extended outbreaks may be connected, and highlighting the diversity of transmission routes that maintain the prevalence of strangles. These transmission routes may include the temporary movement of horses to competitions and shows, sales of horses between different premises and regions, as well as movements and mixing of horses for leisure activities such as hacking and hunting.

When modelling *S. equi* transmission, selection of an appropriate methodology needed to consider within‐host evolution,^15^ slow substitution rates (therefore highly related sequences), low sampling proportion relative to actual cases that are often clinically apparent and presumptively diagnosed without sampling,^11^ and the ability to flexibly test prior assumptions within the model. The combined generation and infection time parameter of 6 weeks (42 ± 10 days) was deemed the most appropriate for sampling/outbreak events in this study, accounting for up to 2 weeks of active infection and 4 weeks of convalescence.^45^ Other parameter values were explored, including the generation time of 13 ± 5 days estimated by Houben et al.^46^ and a much longer period of 182 ± 30 days. The 13 ± 5 days was calculated through published data on outbreaks within single premises, which represented uncontrolled transmission between horses in the same outbreak, when there may have been simultaneous rather than sequential transmission. Furthermore, the third scenario considered transmission events after long‐term carriage (but without considering potential earlier infectious transmission events); the non‐convergence of this scenario was not surprising, given the likely overestimation of this timeframe for our dataset. As such, these assumptions were not considered to be representative of the wider national data and sample collection in this study, where transmission could occur between multiple horse populations from different premises over longer periods.

With the exception of the spatially restricted Dartmoor subcluster of cluster three, other clusters comprised isolates that were recovered from horses more widely across the UK. Given the frequent transport of horses for pleasure, competitions, veterinary care, and buying and selling,^47^ alongside sub‐optimal use of biosecurity measures,^48^ it was not unexpected to see related strains being recovered from multiple regions across the UK over relatively short periods. The 11%–18% sampling proportion achieved within clusters was sufficient to provide insights into the wider transmission of this endemic pathogen through the UK horse population but incurred constraints on our analysis through reliance on the submission of samples to diagnostic laboratories. Our data are consistent with the majority of strangles cases in the UK horse population not being confirmed or perhaps more importantly outbreaks not being controlled by agent detection by diagnostic laboratories, suggesting inadequacies in the diagnosis and clearance of strangles that likely facilitate the continued spread of this pathogen.^11^ To improve sample acquisition for molecular surveillance while simultaneously improving outbreak management and reducing transmission likelihood, it is important to emphasise that veterinary surgeons involved in the management of strangles outbreaks should strongly encourage horse owners to not only seek diagnostic confirmation of the disease but also to carry out post‐infection screening to confirm freedom from infection at the end of the outbreak. Veterinary surgeons are widely regarded as reliable sources of advice by horse owners/keepers.^49^ Therefore, working together with owners to develop a strangles management plan that is practical and financially achievable, but also effective in establishing clearance, is essential in helping to prevent further spread of disease. If cost is a limiting factor, it may be helpful to suggest testing one or two clinically affected horses at the beginning of the outbreak to confirm *S. equi* infection, with this followed by appropriate screening of the wider population, in line with the traffic light outbreak management protocol.^50^ This approach may support and empower owners to take informed and effective steps to manage the outbreak. Encouragingly, where epidemiological information was available, it supported the plausible transmission events identified by Transphylo, suggesting that this could be a useful tool to prospectively monitor the ongoing state of *S. equi* transmission, and when combined with appropriate outbreak data to direct the implementation of new interventions.

Despite only periodic sampling opportunities, the availability of detailed outbreak management history and veterinary information for most animals within the Dartmoor subcluster enabled scrutiny of transmission inference to determine Transphylo's ability to plausibly model *S. equi* transmission. When considering the epidemiological data, the three sub‐strains identified in this subcluster remained within the geographical locations on Dartmoor where ponies were held and sampled during their respective outbreaks. The mixing of highly related sub‐strains within the largest transmission group was observed over two locations, Widecombe and Ponsworthy, which are less than three miles (~5 km) apart by road. The management of Dartmoor ponies enables them to free roam across Dartmoor and they are not restricted to fields by fencing unless held in a new‐take area. Given this management style and the timeline in which transmission has been inferred (2 years before sampling), it is plausible that this subcluster strain of *S. equi* crossed over between the two adjacent herds. Where external transmission links were identified, the epidemiological information available suggested close links to other samples recovered from ponies across Dartmoor.

In cluster three, Transphylo identified an onward transmission chain beginning October 2018 to March 2019, spanning the length of the UK. Within this group of horses, three separate sampled horses were linked via close distances to other sampled horses, and of the four final sampled horses, three were identified by relatively closely located veterinary practices in Scotland. No further epidemiological information was available a priori to confirm this transmission inference, and so it was not known that these isolates were likely to be so closely linked. This small group of highly related and linked sequences provides evidence as to how the movement of horses across the UK facilitates the spread of infectious disease and emphasises the importance of preventative biosecurity, diagnostic screening, and vaccination for disease prevention. Had transmission inference been undertaken closer to the clinical investigations and sampling of horses within this chain, targeted contact tracing and movement logs could theoretically have been scrutinised, enabling the identification of in‐contacts. From there, the application of quarantine/isolation, regular monitoring for clinical signs such as pyrexia, vaccination, and screening protocols for in‐contacts could have been initiated and further transmission halted.

### 4.3 Limitations

A recognised limitation of the surveillance of strangles in the UK is the continued underreporting of clinical history and animal signalment details at the time of submission of diagnostic samples.^11^ Further to that, outbreak management information from affected owners or premises is often limited or unavailable, which impedes the ability to use Transphylo to its full potential. The limited availability of the first sampled bacterial isolates from horses with multiple sampling events may also impact transmission inference. These samples may not be available for several reasons, including owners acquiring a diagnosis in an index case and subsequently not sampling other clinically ill horses until they wish to declare freedom from infection, or bacterial isolates that were not routinely archived as is often the case in commercial diagnostic laboratories outside of collaborative research studies.

Furthermore, Transphylo infers transmission and reconstructs transmission trees based on sampling date and genetic diversity, assuming a transmission bottleneck, whereby only one strain is transmitted from a host to other individuals within the chain.^35^ Multiple strains of *S. equi* have been recovered from horses during the same sampling event.^15^ Therefore, it must be considered that even when a sequenced isolate contributes to transmission analyses, it may not be the complete picture of transmission from a horse co‐infected with multiple strains. In the instance where the direction of transmission between one pair was different between analyses involving a larger cluster and a smaller subcluster, this may be due to a confounding influence of the strains' close genetic relatedness and their identical sampling date. Many of the sequences in the Dartmoor subcluster were recovered as part of batch sampling visits that were the only opportunity to collect samples from this largely free‐roaming, semi‐feral population. Therefore, many sequences had the same sampling date, which was then used within the transmission inference. This bias was unique to this subcluster, and, given the management practices of these horses, the data still help to validate the model for the identification of plausible transmission pairs, although with some ambiguity as to the most likely direction of transmission between the pair of animals with an identified plausible transmission event.

## 5 CONCLUSIONS

The results from this study show that UK *S. equi* strains continue to undergo evolutionary transitions and provide novel contributions to our understanding of *S. equi* transmission dynamics. The population structure of circulating *S. equi* strains was shown to have changed within the relatively short time period (2016–2022) that was investigated, suggesting that the contemporary strains have a significant selective advantage and ability to transmit. Second, our data argue that most detected *S. equi* infections between 2016 and 2022 in the UK were caused by transmission from either cases with overt clinical signs or from recently convalesced subclinical short‐term carrier horses, with transmission of older strains of *S. equi* from long‐term carrier horses having less influence than previously proposed. Our data highlight the importance of greater awareness and adoption of post‐outbreak screening protocols to confirm freedom from infection, rather than owners assuming recovery based on the resolution of clinical signs.

Continued molecular surveillance of *S. equi* strains across the UK (and internationally) is highly recommended to follow the evolutionary path of *S. equi*, which to date has shown a progressive adaptation allowing continued endemicity. By combining epidemiological and genomic data, it has been possible to explore the transmission dynamics of strains between horse populations across the UK. Almost two‐thirds of transmission pairs occurred between different UK regions, and one transmission chain covered large distances across the UK, providing evidence that transmission of *S. equi* via the movements of horses continues to be of national and international importance.^9^ This finding supports the need to improve outbreak management and sampling, surveillance, and prevention of strangles in the UK and elsewhere. If the pipeline from sampling to sequencing to analysis used in this study could be employed in a more real‐time manner, in‐contact animals can be identified, quarantined, and screened, vaccination employed, and the transmission chain halted sooner, thereby preventing the further spread of *S. equi* infection. Additionally, with the ability to identify modes of transmission, intervention measures can be improved, such as implementing temperature checking post‐travel, followed by precautionary isolation and laboratory investigation as appropriate, or administering vaccination as a preventive measure followed by strategic boosting before ‘high‐risk’ events.

## FUNDING INFORMATION

Work undertaken in this study was funded by The Horse Trust.

## CONFLICT OF INTEREST STATEMENT

Andrew S. Waller is CSO at Intervacc. Sara Frosth is funded by Intervacc.

## AUTHOR CONTRIBUTIONS

**Abigail A. McGlennon:** Conceptualization; methodology; data curation; investigation; validation; formal analysis; visualization; project administration; writing – original draft; resources; writing – review and editing. **Kristien L. Verheyen:** Conceptualization; funding acquisition; project administration; supervision; writing – review and editing. **J. Richard Newton:** Conceptualization; funding acquisition; project administration; supervision; writing – review and editing. **Andries van Tonder:** Methodology; software; writing – review and editing. **Hayley Wilson:** Methodology; writing – review and editing. **Julian Parkhill:** Writing – review and editing; methodology. **Nicolas de Brauwere:** Resources; writing – review and editing. **Sara Frosth:** Data curation; methodology; resources; writing – review and editing. **Andrew S. Waller:** Conceptualization; funding acquisition; project administration; supervision; writing – review and editing.

## DATA INTEGRITY STATEMENT

Abigail A. McGlennon takes responsibility for the integrity of the data and the accuracy of the data analysis.

## ETHICAL ANIMAL RESEARCH

Full ethical approval from the Royal Veterinary College's Clinical Research Ethical Review Board was granted (URN 2020 1973‐2).

## INFORMED CONSENT

Not stated.

## Supporting information


**Figure S1.** Network plots with clusters of the pairwise single nucleotide polymorphism (SNP) distances between the 447 S. equi sequences recovered from individual horses between 30 December 2015 and 14 September 2022 across the United Kingdom with the pairwise SNP distance thresholds of one, two, three, 10, 15, and 20 between sequences.

**Figure S2.** Maximum clade credibility phylogenetic tree of 511 S. equi isolates recovered across the United Kingdom between 30 December 2015 and 14 September 2022. The estimated TMRCA for the divergence between the two primary clades is stated with 95% HPD in brackets.

**Table S1.** The origins and details of 511 *Streptococcus equi* sequences recovered from UK horses between 30 December 2015 and 14 September 2022.

**Table S2.** Quality assessment of all 511 *Streptococcus equi* sequenced genomes examined using the BacQC and Bactmap pipelines (https://github.com/avantonder/bacQC, https://nf-co.re/bactmap).

**Table S3.** Different estimated generation and sampling times *of Streptococcus equi* used to explore the effects of these changes on Transphylo's inference of transmission relative to different circumstances of strangles infection and transmission from UK isolates.

**Table S4.** Results of logistic regression analyses investigating associations between sampling year and the recovery of McG‐BAPS types‐3 and‐5 from *S. equi* sequences recovered from UK horses between 4 January 2016 and 14 September 2022. Population groupings were determined by fast hierarchical Bayesian analysis of population structure (fastBAPS).^13^ Coeff = coefficient value, S.E = standard error, OR = odds ratio, 95% CI = 95% confidence intervals. Statistically significant values are in bold.

**Table S5.** Mean Gelman Rubin (GR) diagnostic and effective sample size (ESS) values for the transmission inference parameters of r, pi and neg for each run of a cluster of 16 *S. equi* whole genome sequences testing the prior assumptions generation/sampling time used by the transmission inference package Transphylo.^35^ GR diagnostic values <1.2 and ESS values >100 indicate successful Markov chain Monte Carlo (MCMC) convergence, * = indicating non‐convergence. Scenario 1 = 13 days ±5 (acute transmission), scenario 2 = 42 days ±10 (between outbreak transmission), scenario 3 = 182 days ±30 (transmission from long‐term carriers).

**Table S6.** Mean Gelman Ruben (GR) diagnostic and effective sample size (ESS) values for the transmission inference parameters off.r, pi and neg for clusters of *S. equi* sequences run in triplicate that underwent transmission inference using the R package Transphylo.^35^ Transmission inference was based on the prior assumptions ‘generation time’ (i.e., the time between infection to onward transmission) and ‘sampling time’ (i.e., the time between infection and sampling) being 42 days ±10. GR diagnostic values <1.2 and ESS values >100 indicate successful Markov chain Monte Carlo (MCMC) convergence, * = indicating non‐convergence.

## Data Availability

The data that support the findings will be available at https://github.com/am-epi/sestransmission. Whole genome sequencing data for each isolate will be available in the European Nucleotide Archive (ENA) at EMBL‐EBI under accession numbers PRJEB77744 and PRJEB82429 following an embargo from the date of publication to allow for commercialisation of research findings.
