# Supplementary material for: Unwelcome neighbours: Tracking the transmission of Streptococcus equi in the United Kingdom horse population
Source: Equine Vet J. 2025 Jul 20;58(2):533–48. doi: 10.1111/evj.14558 (PMC12892377; doi:10.1111/evj.14558)
Supplement: Supplementary file 1 — Figure S1. Network plots with clusters of the pairwise single nucleotide polymorphism (SNP) distances between the 447 S. equi sequences recovered from individual horses between 30 December 2015 and 14 September 2022 across the United Kingdom with the pairwise SNP distance thresholds of one, two, three, 10, 15, and 20 between sequences. [file EVJ-58-533-s008.pdf]

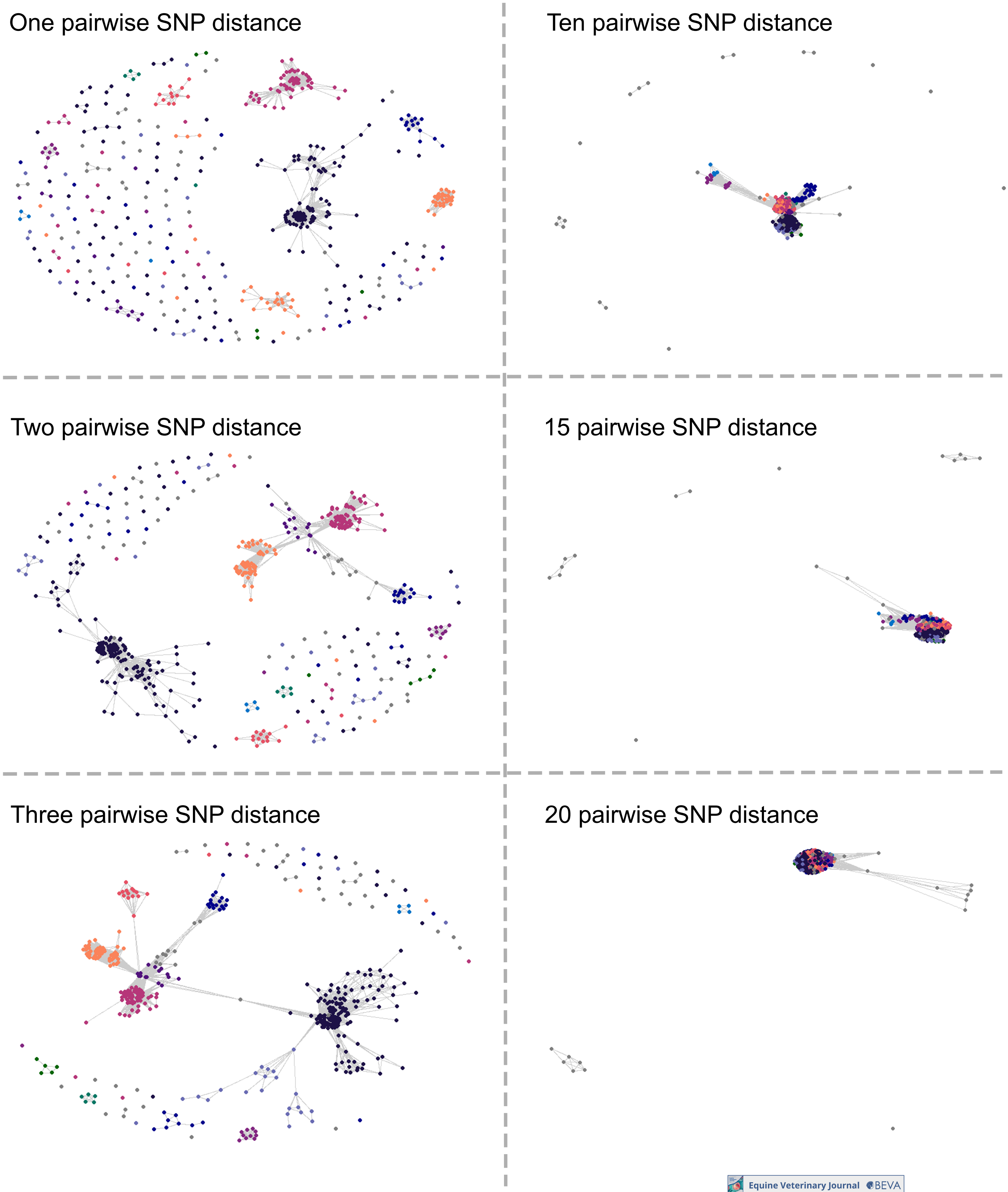

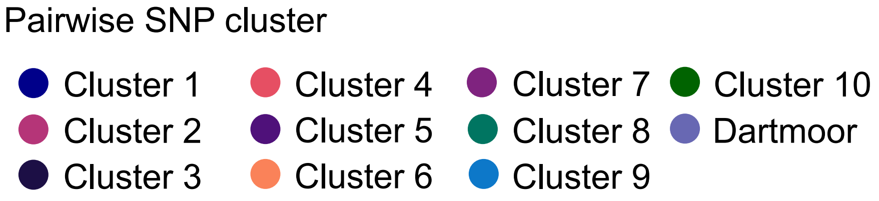

Equine Veterinary Journal BEVA

Figure S1: Network plots with clusters of the pairwise single nucleotide polymorphism (SNP) distances between the 447 S. equi sequences recovered from individual horses between 30 December 2015 and 14 September 2022 across the United Kingdom with the pairwise SNP distance thresholds of one, two, three, 10, 15 and 20 between sequences.
