# Supplementary material for: Unwelcome neighbours: Tracking the transmission of Streptococcus equi in the United Kingdom horse population
Source: Equine Vet J. 2025 Jul 20;58(2):533–48. doi: 10.1111/evj.14558 (PMC12892377; doi:10.1111/evj.14558)
Supplement: Supplementary file 2 — Figure S2. Maximum clade credibility phylogenetic tree of 511 S. equi isolates recovered across the United Kingdom between 30 December 2015 and 14 September 2022. The estimated TMRCA for the divergence between the two primary clades is stated with 95% HPD in brackets. [file EVJ-58-533-s004.pdf]

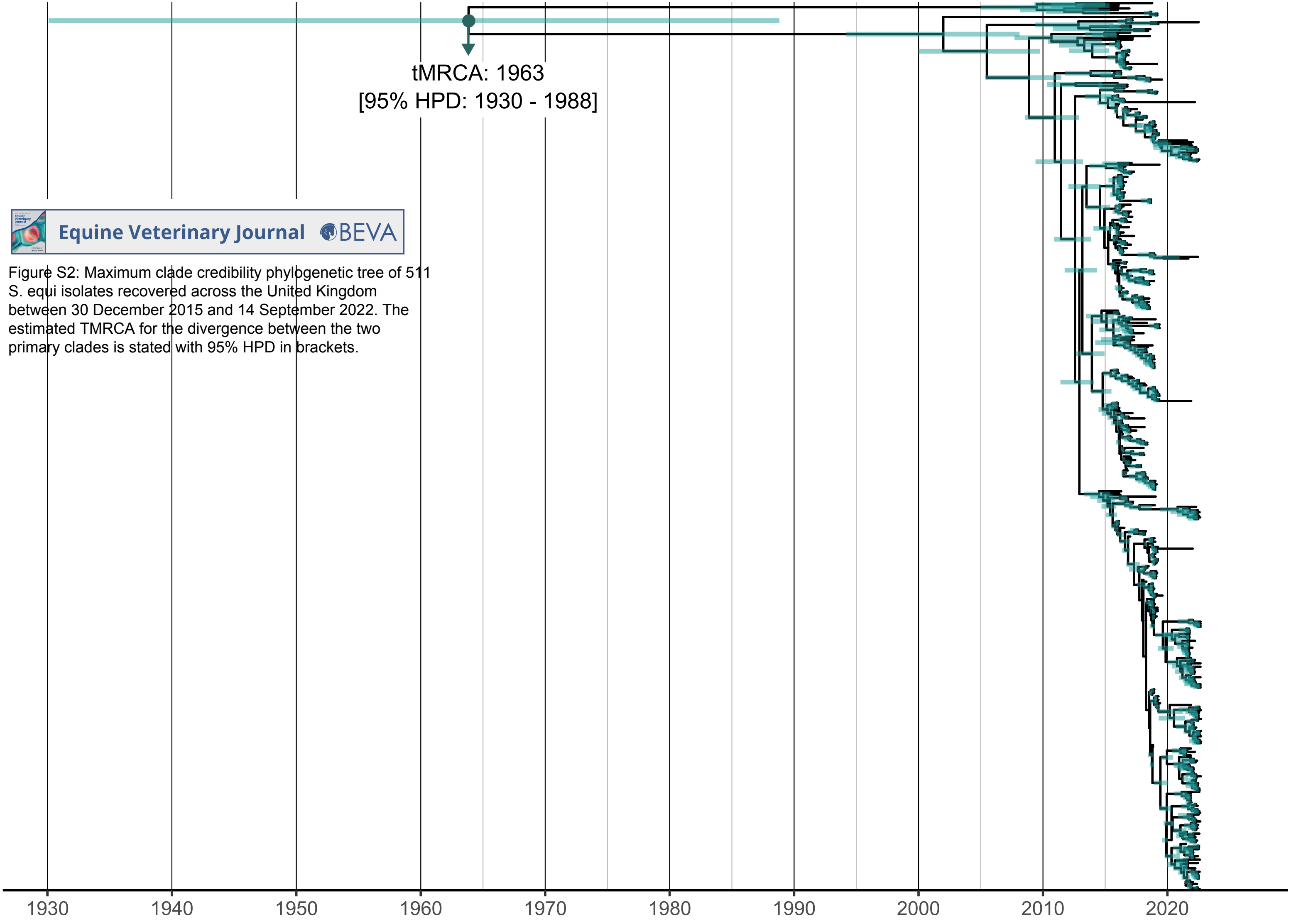

Equine Veterinary Journal BEVA

Figure S2: Maximum clade credibility phylogenetic tree of 511 S. equi isolates recovered across the United Kingdom between 30 December 2015 and 14 September 2022. The estimated TMRCA for the divergence between the two primary clades is stated with 95% HPD in brackets.
