# Supplementary material for: Unwelcome neighbours: Tracking the transmission of Streptococcus equi in the United Kingdom horse population
Source: Equine Vet J. 2025 Jul 20;58(2):533–48. doi: 10.1111/evj.14558 (PMC12892377; doi:10.1111/evj.14558)
Supplement: Supplementary file 5 — Table S3. Different estimated generation and sampling times of Streptococcus equi used to explore the effects of these changes on Transphylo's inference of transmission relative to different circumstances of strangles infection and transmission from UK isolates. [file EVJ-58-533-s005.pdf]

Table S3: Different estimated generation and sampling times of *Streptococcus equi* used to explore the effects of these changes to Transphylo's inference of transmission relative to different circumstances of strangles infection and transmission from UK isolates.

Scenario	Generation / sampling time (days)	Deviation (days)	Justification
**1**	13	5	Generation time reported in the literature used to estimate the R_0 number of *S. equi* (Houben *et al.*, 2022).
**2**	42	10	Accounting for an average of two weeks of active infection and four weeks after the cessation of clinical signs (Rendle *et al.*, 2021) [45].
**3**	182	30	Accounting for developing into a long-term carrier of *S. equi* for six months.
