# Supplementary material for: Unwelcome neighbours: Tracking the transmission of Streptococcus equi in the United Kingdom horse population
Source: Equine Vet J. 2025 Jul 20;58(2):533–48. doi: 10.1111/evj.14558 (PMC12892377; doi:10.1111/evj.14558)
Supplement: Supplementary file 6 — Table S4. Results of logistic regression analyses investigating associations between sampling year and the recovery of McG‐BAPS types‐3 and‐5 from S. equi sequences recovered from UK horses between 4 January 2016 and 14 September 2022. Population groupings were determined by fast hierarchical Bayesian analysis of population structure (fastBAPS). 13 Coeff = coefficient value, S.E = standard error, OR = odds ratio, 95% CI = 95% confidence intervals. Statistically significant values are in bold. [file EVJ-58-533-s006.pdf]

Equine Veterinary Journal BEVA

Table S4: Results of logistic regression analyses investigating associations between sampling year and the recovery of McG-BAPS types -3 and -5 from *S. equi* sequences recovered from UK horses between 4 January 2016 and 14 September 2022. Population groupings were determined by fast hierarchical Bayesian analysis of population structure (fastBAPS) [13]. Coeff = coefficient value, S.E = standard error, OR = odds ratio, 95%CI = 95% confidence intervals. Statistically significant values are in bold.

**McG-BAPS3:**	**Negative** (n= 247)		**Positive** (n= 199)		**Ordinary logistic regression**				
**Year**	**n**	**%**	**n**	**%**	**Coeff.**	**S.E.**	**OR**	**OR 95%CI**	**Wald χ^2 P-value**
2016	99	92.5	8	7.5	ref		1		
2017	42	91.3	4	8.7	0.164	0.640	1.18	0.3 - 3.9	0.79
2018	61	68.5	28	31.5	1.737	0.433	5.68	2.53 - 14.09	**<0.001**
2019	28	51.9	26	48.1	2.442	0.458	11.49	4.87 - 29.81	**<0.001**
2021	8	20	32	80	3.902	0.540	49.50	18.23 - 153.56	**<0.001**
2022	9	8.2	101	91.8	4.934	0.506	138.88	54.88 - 404.16	**<0.001**
**McG-BAPS5:**	**Negative** (n= 279)		**Positive** (n= 167)		**Ordinary logistic regression**				
**Year**	**n**	**%**	**n**	**%**	**Coeff.**	**S.E.**	**OR**	**OR 95%CI**	**Wald χ^2 P-value**
2016	37	34.6	70	65.4	ref		1		
2017	15	32.6	31	67.4	0.088	0.374	1.09	0.53 - 2.31	0.81
2018	42	47.2	47	52.8	-0.525	0.294	0.59	0.33 - 1.05	0.07
2019	38	70.4	16	26.9	-1.503	0.361	0.22	0.11 - 0.44	**<0.001**
2021	38	95	2	5	-3.582	0.753	0.03	0 - 0.09	**<0.001**
2022	109	99.1	1	0.1	-5.329	1.025	0.00	0 - 0.02	**<0.001**
