# Supplementary material for: Unwelcome neighbours: Tracking the transmission of Streptococcus equi in the United Kingdom horse population
Source: Equine Vet J. 2025 Jul 20;58(2):533–48. doi: 10.1111/evj.14558 (PMC12892377; doi:10.1111/evj.14558)
Supplement: Supplementary file 7 — Table S5. Mean Gelman Rubin (GR) diagnostic and effective sample size (ESS) values for the transmission inference parameters of r, pi and neg for each run of a cluster of 16 S. equi whole genome sequences testing the prior assumptions generation/sampling time used by the transmission inference package Transphylo. 35 GR diagnostic values <1.2 and ESS values >100 indicate successful Markov chain Monte Carlo (MCMC) convergence, * = indicating non‐convergence. Scenario 1 = 13 days ±5 (acute transmission), scenario 2 = 42 days ±10 (between outbreak transmission), scenario 3 = 182 days ±30 (transmission from long‐term carriers). [file EVJ-58-533-s003.pdf]

Equine Veterinary Journal BEVA

Table S5: Mean Gelman Ruben (GR) diagnostic and effective sample size (ESS) values for the transmission inference parameters off.r, pi and neg for each run of a cluster of 16 *S.equi* whole genome sequences testing the prior assumptions generation/sampling time used by the transmission inference package Transphylo [35]. GR diagnostic values < 1.2 and ESS values > 100 indicate successful Markov chain Monte Carlo (MCMC) convergence, * = indicating non-convergence. (scenario 1 = 13 days ± 5 (acute transmission), scenario 2 = 42 days ± 10 (between outbreak transmission), scenario 3 = 182 days ± 30 (transmission from long-term carriers).

	off.r			pi			neg		
**Scenario**	**ESS**	**mean**	**95% Credible Interval**	**ESS**	**mean**	**95% Credible Interval**	**ESS**	**mean**	**95% Credible Interval**
**1**	66401	1.01	0.97, 1.05	5268	0.0103	0.0100, 0.0113	2520	1	0.03, 3.59
**2**	87115	1.04	0.95, 1.13	48320	0.0156	0.0102, 0.0266	2054	1.01	0.04, 3.72
**3**	44034	1.21*	0.81, 1.69	8614	0.289	0.1400, 0.4790	1866	0.48	0.09, 2.43
