# Supplementary material for: Unwelcome neighbours: Tracking the transmission of Streptococcus equi in the United Kingdom horse population
Source: Equine Vet J. 2025 Jul 20;58(2):533–48. doi: 10.1111/evj.14558 (PMC12892377; doi:10.1111/evj.14558)
Supplement: Supplementary file 8 — Table S6. Mean Gelman Ruben (GR) diagnostic and effective sample size (ESS) values for the transmission inference parameters off.r, pi and neg for clusters of S. equi sequences run in triplicate that underwent transmission inference using the R package Transphylo. 35 Transmission inference was based on the prior assumptions ‘generation time’ (i.e., the time between infection to onward transmission) and ‘sampling time’ (i.e., the time between infection and sampling) being 42 days ±10. GR diagnostic values <1.2 and ESS values >100 indicate successful Markov chain Monte Carlo (MCMC) convergence, * = indicating non‐convergence. [file EVJ-58-533-s001.pdf]

Equine Veterinary Journal BEVA

Table S6: Mean Gelman Ruben (GR) diagnostic and effective sample size (ESS) values for the transmission inference parameters off.r, pi and neg for clusters of S.equi sequences run in triplicate that underwent transmission inference using the R package Transphylo [35]. Transmission inference was based on the prior assumptions 'generation time' (i.e., the time between infection to onward transmission) and 'sampling time' (i.e., the time between infection and sampling) being 42 days ± 10. GR diagnostic values < 1.2 and ESS values > 100 indicate successful Markov chain Monte Carlo (MCMC) convergence, * = indicating non-convergence.

	off.r			pi			neg		
	**ESS**	**mean**	**95% Credible Interval**	**ESS**	**mean**	**95% Credible Interval**	**ESS**	**mean**	**95% Credible Interval**
Cluster one - run one	84675	1.05	0.98, 1.14	26323	0.0187	0.0112, 0.0296	1792	1.19	0.11, 4.06
Cluster one - run two	83203	1.05	0.97, 1.13	31678	0.0188	0.0112, 0.0296	2336	1.20*	0.14, 3.83
Cluster one - run three	83282	1.05	0.97, 1.13	30421	0.0188	0.0112, 0.0297	2323	1.11	0.06, 3.74
Cluster two - run one	72198	1.01	0.96, 1.06	27573	0.0204	0.0143, 0.0277	2103	1.08	0.09, 3.75
Cluster two - run two	71499	1.01	0.96, 1.06	28333	0.0206	0.0144, 0.0279	2188	1.22*	0.15, 3.97
Cluster two - run three	69031	1.01	0.96, 1.06	28350	0.0206	0.0145, 0.0281	1997	1.30*	0.18, 4.22
Cluster three - run one	65368	1.1	1.06, 1.15	10663	0.0299	0.0238, 0.0369	870	0.97	0.11, 3.48
Cluster three - run two	56097	1.1	1.06, 1.15	12144	0.0294	0.0234, 0.0363	719	0.39	0.06, 2.20
Cluster three - run three	46476	1.1	1.06, 1.15	9533	0.029	0.0231, 0.0358	962	0.19	0.04, 1.28
Dartmoor - run one *(Cluster three subgroup)*	80416	1.16	1.05, 1.28	9573	0.0199	0.0109, 0.0342	547	0.38	0.002, 2.69
Dartmoor - run two *(Cluster three subgroup)*	63717	1.16	1.05, 1.28	15054	0.019	0.0108, 0.0325	783	0.02	0.002, 0.04
Dartmoor - run three *(Cluster three subgroup)*	60257	1.16	1.05, 1.28	11257	0.0202	0.0110, 0.0352	580	0.46	0.008, 2.9
Cluster four - run one	68551	1.20*	1.02, 1.40	30487	0.0334	0.0143, 0.0639	2161	0.82	0.02, 3.23
Cluster four - run two	68054	1.20*	1.02, 1.40	23164	0.033	0.0141, 0.0628	1402	0.65	0.01, 3.18
Cluster four - run three	60326	1.20*	1.03, 1.40	28016	0.0331	0.0140, 0.0631	1751	0.81	0.02, 3.53
Cluster five - run one	87595	1.04	0.95, 1.13	43427	0.0156	0.0102, 0.0268	1699	0.92	0.02, 3.40
Cluster five - run two	85738	1.04	0.95, 1.13	44202	0.0156	0.0102, 0.0268	2147	1.06	0.06, 3.74
Cluster five - run three	86911	1.04	0.95, 1.13	49660	0.0157	0.0102, 0.0268	2084	1.13	0.07, 4.18

Cluster six - run one	77027	1.01	0.95, 1.07	17639	0.0266	0.0180, 0.0372	970	0.92	0.03, 4.05
Cluster six - run two	78073	1.01	0.95, 1.07	26848	0.0296	0.0183, 0.0374	2411	1.16	0.13, 3.74
Cluster six - run three	78196	1.01	0.95, 1.07	27191	0.0269	0.0184, 0.0375	1765	1.15	0.14, 3.87
Cluster seven - run one	83654	1.04	0.87, 1.23	22322	0.0437	0.0173, 0.0860	1856	0.98	0.02, 3.70
Cluster seven - run two	83847	1.04	0.87, 1.23	25970	0.0436	0.0175, 0.0856	1238	0.98	2.75, 3.91
Cluster seven - run three	84066	1.04	0.87, 1.23	30895	0.0436	0.0173, 0.0850	2294	0.98	0.04, 3.59
